# Endovascular Therapy and Outcomes Among Patients With Very Large Ischemic Core Stroke

**DOI:** 10.1001/jamanetworkopen.2024.9298

**Published:** 2024-05-02

**Authors:** Dongjing Xie, Jiacheng Huang, Shitao Fan, Changwei Guo, Wenzhe Sun, Zhouzhou Peng, Lingyu Zhang, Chengsong Yue, Zhongming Qiu, Hongfei Sang, Dingwen Liang, Jinrong Hu, Jie Yang, Jiandi Huang, Linyu Li, Juan Liu, Dahong Yang, Xiang Liu, Weilin Kong, Shuai Liu, Qingwu Yang, Wenjie Zi, Fengli Li

**Affiliations:** 1 Department of Neurology, Xinqiao Hospital and The Second Affiliated Hospital, Army Medical University (Third Military Medical University), Chongqing, China; 2 Department of Neurology, Weifang Medical University, Weifang, China; 3 Department of Neurology, Affiliated Hangzhou First People’s Hospital, Zhejiang University School of Medicine, Hangzhou, China; 4 Department of Neurology, Southwest Hospital, Army Medical University (Third Military Medical University), Chongqing, China

## Abstract

**Importance:**

The association of endovascular therapy (EVT) with outcomes is unclear for patients with very low Alberta Stroke Program Early Computed Tomography Score (ASPECTS) within 24 hours of stroke onset.

**Objective:**

To explore the association of EVT with functional and safety outcomes among patients with ASPECTS of 0 to 2 scored with noncontrast computed tomography.

**Design, Setting, and Participants:**

This cohort study used data from an ongoing, prospective, observational, nationwide registry including all patients treated at 38 stroke centers in China with an occlusion in the internal carotid artery or M1 or M2 segment of the middle cerebral artery within 24 hours of witnessed symptom onset. Patients with ASPECTS of 0 to 2 between November 1, 2021, and February 8, 2023, were included in analysis. Data were analyzed October to November 2023.

**Exposures:**

EVT vs standard medical treatment (SMT).

**Main Outcomes and Measures:**

The primary outcome was favorable functional outcome, defined as modified Rankin Scale score (mRS) of 0 to 3, at 90 days. Safety outcomes included symptomatic intracerebral hemorrhage (sICH) within 48 hours and mortality at 90 days.

**Results:**

A total of 245 patients (median [IQR] age, 71 [63-78] years; 118 [48%] women) with ASPECTS of 0 to 2 were included, of whom 111 patients (45.1%) received SMT and 135 patients (54.9%) received EVT. The EVT group had significantly greater odds of favorable functional outcome at 90 days than the SMT group (30 patients [22.2%] vs 11 patients [9.9%]; *P* = .01; adjusted odds ratio [aOR], 3.07 [95% CI, 1.29-7.31]; *P* = .01). Patients in the EVT group, compared with the SMT group, had significantly greater odds of any ICH (56 patients [41.5%] vs 16 patients [11.4%]; *P* < .001; aOR, 4.27 [95% CI, 2.19-8.35]; *P* < .001) and sICH (24 patients [17.8%] vs 1 patient [0.9%]; *P* < .001; aOR, 23.07 [95% CI, 2.99-177.79]; *P* = .003) within 48 hours. There were no differences between groups for 90-day mortality (80 patients [59.3%] vs 59 patients [53.2%]; *P* = .34; aOR, 1.38 [95% CI, 0.77-2.47]; *P* = .28). The results remained robust in the propensity score–matched analysis.

**Conclusions and Relevance:**

In this cohort study of patients with very low ASPECTS based on NCCT within 24 hours of stroke onset, those treated with EVT had higher odds of a favorable functional outcome compared with those who received SMT. Randomized clinical trials are needed to assess these findings.

## Key Points

**Question:**

What is the association of endovascular therapy (EVT) vs standard medical treatment with outcomes in patients with very large ischemic core stroke?

**Findings:**

In this cohort study of 245 patients with Alberta Stroke Program Early Computed Tomography Score of 0 to 2 based on noncontrast computed tomography findings within 24 hours of stroke onset, EVT was associated with higher proportion of favorable functional outcome at 90 days compared with standard medical treatment alone.

**Meaning:**

These findings suggest that EVT should be considered a useful strategy for treatment of patients with very large ischemic core.

## Teaser

This cohort study explores outcomes associated with endovascular therapy vs standard medical treatment alone for patients with very large ischemic core stroke, as assessed with noncontrast computed tomography.

## Introduction

Recently, a few randomized clinical trials (RCTs) have explored the effect of endovascular treatment (EVT) in patients with low Alberta Stroke Program Early Computed Tomography Score (ASPECTS) and found significant benefit of EVT in patients with large vessel anterior circulation strokes and a large infarct core compared with standard medical treatment (SMT)^1,2,3,4^; although the Thrombectomy for Emergent Salvage of Large Anterior Circulation Ischemic Stroke trial did not find significant benefit.^5^ However, there were few studies focusing on the patients with very large infarct (ASPECTS, 0-2). In patients with large ischemic core, especially those with very large infarct, there may be existent penumbra that could be salvaged through EVT.

In a systematic review and meta-analysis^6^ of subgroups of patients with baseline ASPECTS of 0 to 2 included in the ANGEL-ASPECT trial^2^ and SELECT2 trial,^1^ a statistically significant shift in the distribution of modified Rankin Scale (mRS) scores toward better outcomes in favor of EVT was observed in the pooled analyses. In the RESCUE-Japan LIMIT trial,^7^ EVT was not associated with improved 90-day functional outcomes in patients with ASPECTS of 3 or less. Limited sample size and different imaging selection methods among those trials limit the value of the systematic review. The enrolled patients of previous RCTs were screened mainly by advanced imaging with computed tomography perfusion or magnetic resonance imaging (MRI). But advanced imaging selection may cause delay for treatment and exclude the patients that could benefit from EVT.^8^ Inversely, noncontrast computed tomography (NCCT) is available at all stroke centers in clinical practice in China. There were no significant differences observed in the clinical outcomes of patients selected with NCCT compared with those selected with advanced imaging in previous studies.^9,10^ Therefore, the aim of our study was to explore the association of EVT for patients with very large ischemic core, ie patients with ASPECTS of 0 to 2, scored with NCCT in a nationwide registry of acute anterior circulation large artery occlusion in medical practice.

## Methods

### Study Design

This protocol for the registry was approved by ethics committee of the Second Affiliated Hospital (Xinqiao Hospital) of the Army Medical University and the institutional review boards at each of the included centers. All enrolled patients or their legally authorized representatives provided written informed consent before enrollment. The study followed the Strengthening the Reporting of Observational Studies in Epidemiology (STROBE) reporting guideline for cohort studies. Data were collected from an ongoing, prospective, observational, nationwide registry including all patients from 38 stroke centers across China with an occlusion in the internal carotid artery or M1 or M2 segment of the middle cerebral artery within 24 hours of witnessed symptom onset between November 1, 2021, and February 8, 2023.

The inclusion criteria for this analysis were age at least 18 years; acute ischemic stroke due to anterior circulation large vessel occlusion, defined as occlusion of the internal carotid artery or the M1 segment or M2 segment of the middle cerebral artery; large ischemic core identified on NCCT (defined as an ASPECTS of 0-2); and symptom presentation within 24 hours (the time metric of time-last-known-well within 24 hours was used instead if presentation time was unavailable). Patients were excluded from the study if they had prestroke mRS score greater than 2, lacked of follow-up information on 90-day outcomes, or had serious or terminal illness that was not related to ischemic stroke. Patients were divided into SMT and EVT plus SMT groups. The ASPECTS of all patients was scored with NCCT.

### Data Collection and Clinical Outcome

The primary outcome measure was favorable functional outcome, defined as a score of 0 to 3 on the mRS, which ranges from 0 (no symptoms) to 6 (death), at 90 days. Secondary outcomes included functional independence (mRS score, 0-2), excellent functional outcome (mRS score, 0-1), and an ordinal shift across the range of mRS scores toward a better outcome at 90 days. Safety outcomes included any intracranial hemorrhage (ICH) and symptomatic ICH (sICH) according to the Heidelberg Bleeding Classification within 48 hours after admission,^11^ cerebral hernia during hospitalization, and mortality rate at 90 days.

### Radiologic Assessment

The imaging core laboratory evaluated the findings on baseline NCCT for the ASPECTS, baseline vessel imaging (CT angiography, MRI angiography, or digital subtraction angiography) for the location of the occlusion, angiographic outcomes on digital subtraction angiography imaging for technical efficacy outcomes regarding reperfusion, follow-up CT angiography or MRI angiography within 48 hours for vessel recanalization, and the follow-up CT for the presence of ICH. All neuroimaging findings were evaluated independently by 2 neuroradiologists who were unaware of the treatment group assignments, clinical data, and outcomes. For instances of disagreement, decisions were made by a third experienced neuroradiologist.

### Statistical Analysis

Descriptive statistics were used to report patient demographic and clinical characteristics. We used median and IQR values for continuous variables and numbers and percentages for categorical variables. Based on a post hoc power analysis, the current sample size would provide a power of 0.904 with a 2-side α = .05 (eMethods in Supplement 1). The characteristics of the 2 groups were compared using Mann-Whitney *U* test for continuous variables (median and IQR) and the χ^2^ test or Fisher exact test for categorical variables (percentages).

The association between EVT and clinical outcomes in patients with ASPECTS of 0 to 2 was assessed by using univariate and multivariate logistic regression. In multivariate logistic regression model, the adjusted confounding factors included age, sex, baseline National Institutes of Health Stroke Scale (NIHSS) score, intravenous thrombolysis (IVT), last-seen-well to imaging time (onset to imaging), occlusion site, hypertension, systolic blood pressure, and stroke causative mechanism. We performed shift analysis on mRS score using ordinal logistic regression analysis. The proportional odds assumption was tested using approximate likelihood-ratio test of proportionality of odds.

For sensitivity analysis, propensity score matching (PSM) methods were used to balance prognostic important factors. The propensity score was estimated using a multivariable logistic regression model, with the treatment received as the dependent variable and sex, age, history of atrial fibrillation, hypertension, smoking, hyperlipidemia, diabetes, systolic blood pressure, diastolic blood pressure, IVT, baseline NIHSS score, baseline ASPECTS, stroke causative mechanism, occlusion sites, hemisphere, and onset-to-imaging time as covariates. We performed a 1:1 matching based on the nearest-neighbor matching with a 0.2 caliper.

We further investigated the heterogeneity in treatment effect size for the primary outcome within the following subgroups: age (<70 vs ≥70 years), baseline NIHSS score (<18 vs ≥18), IVT (no vs yes), stroke causative mechanism, time from last-known-well to imaging (<360 vs ≥360 minutes). A multiplicative term was entered into regression models to estimate the significance of the interaction with the treatment assignment.

Statistical analyses were performed using SPSS version 23 (IBM) and RStudio software version 1.3.1093 (R Project for Statistical Computing). A 2-tailed *P* < .05 was considered statistically significant. Data were analyzed from October to November 2023.

## Results

### Baseline Characteristics

A total of 245 eligible patients with ASPECTS of 0 to 2 (median [IQR] age, 71 [63-78] years; 118 [48%] women) were selected. Among these patients, 135 patients received EVT and 111 patients were treated with SMT.

The SMT group had higher systolic blood pressure than the EVT group (median [IQR] 154 [134-180] mm Hg vs 147 [130-164] mm Hg; *P* = .006). Additionally, the SMT group, compared with the EVT group, had a higher proportion of M1 middle cerebral artery segment occlusion (67 patients [60.4%] vs 44 patients [32.6%]) and lower proportion of intracranial internal carotid artery occlusion (41 patients [36.9%] vs 79 patients [58.5%]) and M2 middle cerebral artery segment occlusion (3 patients [2.7%] vs 12 patients [8.9%]) (*P* < .001). There were no significant differences between groups in age, diabetes, atrial fibrillation, hyperlipidemia, smoking, diastolic blood pressure, stroke, hemisphere, glucose, IVT, anesthesia, baseline NIHSS score, onset-to-imaging time, and stroke causative mechanism (Table 1).

**Table 1.** Baseline Patient Characteristics

Characteristic	Patients, No. (%)			*P* value
	All	SMT (n = 111)	EVT (n = 135)	
Age, median (IQR), y	71 (63-78)	72 (65-78)	70 (59-77)	.12
Sex				
Female	118 (48.0)	52 (46.8)	66 (48.9)	.08
Male	128 (52.0)	59 (53.2)	69 (51.1)	
Medical history				
Diabetes	42 (17.1)	20 (18.0)	22 (16.3)	.72
Hypertension	151 (61.4)	75 (67.6)	76 (56.3)	.07
Atrial fibrillation	117 (47.6)	57 (51.4)	60 (44.4)	.28
Hyperlipidemia	43 (17.5)	16 (14.4)	27 (20.0)	.25
Smoking	57 (23.2)	26 (23.4)	31 (23.0)	.93
Clinical characteristics				
Blood pressure, median (IQR), mm Hg				
Systolic	150 (133-170)	154 (134-180)	147 (130-164)	.006
Diastolic	87 (76-100)	88 (77-102)	85 (73-100)	.24
Hemisphere				
Left	118 (48)	56 (50.5)	62 (45.9)	.48
Right	128 (52)	55 (49.5)	73 (54.1)	
Glucose, median (IQR), mmol/L^a^	7.2 (6.1-8.7)	7.1 (6.0-8.6)	7.2 (6.2-8.9)	.39
IVT	62 (25.2)	30 (27.0)	32 (23.7)	.55
General anesthesia	NA	NA	23 (17.0)	NA
NIHSS score, median (IQR)	18 (15-23)	18 (14-25)	18 (15-22)	.98
ASPECTS				
0-1	157 (63.8)	83 (74.8)	74 (54.8)	.001
2	89 (36.2)	28 (25.2)	61 (45.2)	
Onset to imaging time, median (IQR), min^b^	305 (162.5-504.0)	314 (168.5-611.3)	295 (152.0-455.0)	.18
Onset to puncture time, median (IQR), min^c^	NA	NA	353.5 (234.5-535.8)	NA
Onset to recanalization time, median (IQR), min^d^	NA	NA	439.5 (323.8-615.0)	NA
Occlusion site				
Intracranial internal carotid artery	120 (48.8)	41 (36.9)	79 (58.5)	<.001
M1 middle cerebral artery segment	111 (45.1)	67 (60.4)	44 (32.6)	
M2 middle cerebral artery segment	15 (6.1)	3 (2.7)	12 (8.9)	
Stroke causative mechanism				
LAA	77 (31.3)	41 (36.9)	36 (26.7)	.24
Cardioembolism	137 (55.7)	58 (52.3)	79 (58.5)	
Other	9 (3.7)	2 (1.8)	7 (5.2)	
Unknown	23 (9.3)	10 (9.0)	13 (9.6)	
Successful recanalization (mTICI ≥2b)	NA	NA	110 (81.5)	NA

Abbreviations: ASPECTS, Alberta Stroke Program Early Computed Tomography Score; EVT, endovascular treatment; IVT, intravenous thrombolysis; LAA, large artery atherosclerosis; mTICI, modified Thrombolysis In Cerebral Infarction score; NIHSS, National Institutes of Health Stroke Scale; SMT, standard medical treatment.

^a^ Data on glucose were missing for 4 patients in the EVT group and 3 patients in the SMT group.

^b^ Data on onset to imaging were missing for 5 patients in the SMT group.

^c^ Data on onset to puncture were missing for 1 patient in the EVT group.

^d^ Data on onset to recanalization were missing for 1 patient in the EVT group.

There were 70 people in each treatment group after the PSM analysis. Baseline characteristics between PSM groups were generally balanced (eTable 1 in Supplement 1).

### Primary Outcome

The EVT group had a significantly higher proportion of patients with favorable functional outcome at 90 days compared with the SMT group (30 patients [22.2%] vs 11 patients [9.9%]; *P* = .01; adjusted odds ratio [aOR], 3.07 [95% CI, 1.29-7.31]; *P* = .01) (Table 2). After PSM, the proportion of patients with favorable functional outcome remained significantly higher in the EVT group compared with the SMT group (24.3% vs 8.6%; *P* = .01; aOR, 5.00 [95% CI, 1.63-15.27]; *P* = .005) (eTable 2 in Supplement 1).

**Table 2.** Primary, Secondary, and Safety Outcomes Associated With EVT vs SMT Among Patients With Very Large Ischemic Core Stroke

Outcomes	Patients, No. (%)		*P* value	Unadjusted		Adjusted^a^	
	SMT (n = 111)	EVT (n = 135)		OR	*P* value	OR	*P* value
Primary outcome: mRS score 0-3	11 (9.9)	30 (22.2)	.01	2.60 (1.24-5.46)	.01	3.07 (1.29-7.31)	.01
Secondary outcome: mRS score							
0-1	1 (0.9)	4 (3.0)	.25	3.36 (0.37-30.49)	.28	4.72 (0.40-55.43)	.22
0-2	2 (1.8)	18 (13.0)	.001	8.39 (1.90-36.98)	.005	35.32 (3.43-364.13)	.003
5	16 (14.4)	8 (5.9)	.03	0.37 (0.15-0.91)	.03	0.38 (0.15-0.96)	.04
Safety outcomes							
sICH	1 (0.9)	24 (17.8)	<.001	23.78 (3.16-178.88)	.002	23.07 (2.99-177.79)	.003
Any ICH	16 (11.4)	56 (41.5)	<.001	4.21 (2.24-7.91)	<.001	4.27 (2.19-8.35)	<.001
Cerebral hernia	22 (19.8)	63 (46.7)	<.001	3.54 (1.99-6.30)	<.001	3.49 (1.83-6.63)	<.001
Mortality	59 (53.2)	80 (59.3)	.34	1.28 (0.77-2.13)	.34	1.38 (0.77-2.47)	.28

Abbreviations: EVT, endovascular treatment; ICH, intracerebral hemorrhage; OR, odds ratio; sICH, symptomatic intracerebral hemorrhage; SMT, standard medical treatment.

^a^ Adjusting for age, sex, baseline National Institutes of Health Stroke Scale score, intravenous thrombolysis, onset to imaging time, occlusion site, hypertension, systolic blood pressure, and stroke causative mechanism.

### Secondary Outcomes

**Figure 1.**
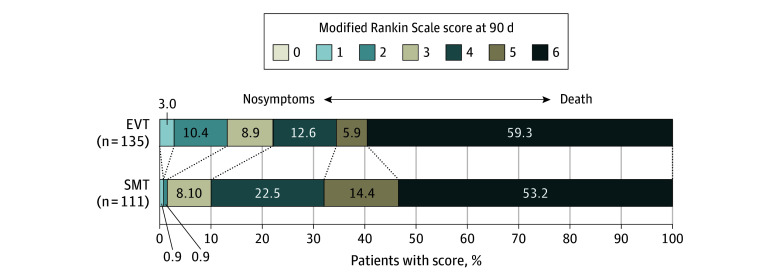
Distribution of the Modified Rankin Scale Score at 90 Days EVT indicates endovascular treatment; SMT, standard medical treatment.

Patients in the EVT group were significantly more likely to achieve functional independence at 90 days compared with the SMT group (18 patients [13.0%] vs 2 patients [1.8%]; *P* = .001; aOR, 35.32 [95% CI, 3.43-364.13]; *P* = .003) (Table 2). There was no statistically significant difference for excellent functional outcome (Table 2). The proportion of patients with mRS score of 5 in the EVT group was lower than in the SMT group (16 patients [14.4%] vs 8 patients [5.9%]; *P* = .03; aOR, 0.38 [95% CI, 0.15-0.96]; *P* = .04). The shift analysis of mRS was not statistically different between groups (adjusted common OR, 1.14 [95% CI, 0.68-1.93]; *P* = .62); however, the proportional odds assumption was significantly violated, indicating that the estimated common OR was not reliable. (*P* for proportional odds assumption <.001) (Figure 1). Similar secondary outcomes were observed after PSM (eTable 2 and eFigure 1 in Supplement 1).

### Safety Outcomes

The occurrence of sICH within 48 hours was higher in the EVT group than in the SMT group (24 patients [17.8%] vs 1 patient [0.9%]; *P* < .001; aOR, 23.07 [95% CI, 2.99-177.79]; *P* = .003). There was no difference between the EVT and SMT groups in 90-day mortality (80 patients [59.3%] vs 59 patients [53.2%]; *P* = .34; aOR, 1.38 [95% CI, 0.77-2.47]; *P* = .28) (Table 2).

The EVT group had higher risk of any ICH than the SMT group (56 patients [41.5%] vs 16 patients [11.4%]; *P* < .001; aOR, 4.27 [95% CI, 2.19-8.35]; *P* < .001). The proportion of patients with cerebral hernia was higher in the EVT group than the SMT group (63 patients [46.7%] vs 22 patients [19.8%]; *P* < .001; aOR, 3.49 [95% CI, 1.83-6.63]; *P* < .001) (Table 2).

### Subgroup Analyses

**Figure 2.**
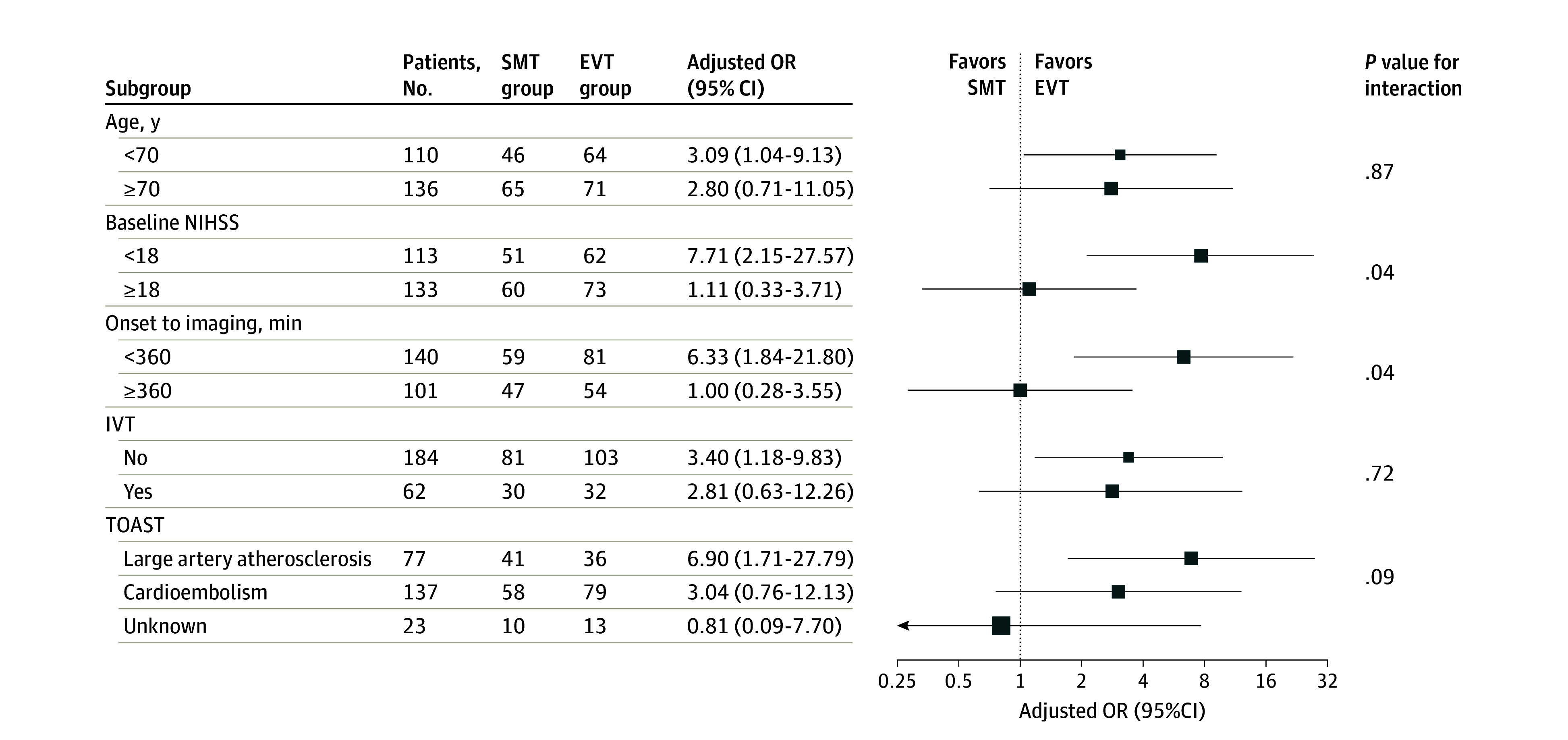
Subgroup Analysis of the Association of Endovascular Treatment (EVT) vs Standard Medical Treatment (SMT) With a Favorable Functional Outcome Among Patients With Very Large Ischemic Core Stroke Favorable functional outcome was defined as modified Rankin Scale score of 0 to 3. IVT indicates intravenous thrombolysis; NIHSS, National Institutes of Health Stroke Scale; OR, odds ratio; and TOAST, Trial of Org 10172 in Acute Stroke Treatment.

Subgroup analyses were performed according to baseline characteristics to explore the association of EVT in patients with ASPECTS of 0 to 2. The results of the subgroup analyses are shown in Figure 2. The results of subgroup analyses to determine the potential heterogeneity of the treatment outcome according to baseline covariates suggested that the benefit of EVT over SMT may have been greater among patients with lower baseline NIHSS score and shorter onset-to-imaging time.

## Discussion

In this cohort study conducted in China, patients with ASPECTS of 0 to 2 scored with NCCT had greater odds of favorable functional outcome at 90 days after EVT within 24 hours after stroke compared with those treated with SMT alone. The occurrence of sICH within 48 hours was higher in the EVT group than in the SMT group, while there were no statistical differences in mortality between groups.

In the subgroup analyses of the LASTE trial, the shift of mRS ordinal categories of patients with ASPECTS of 0 to 2 favored the EVT group.^12^ However, our study enrolled patients within 24 hours of witnessed symptoms and the median time of onset to recanalization was approximately 7 hours, which exceeded the inclusion criteria of less than 6.5 hours since last-known-well time in the LASTE trial.^12^ A systematic review and meta-analysis^6^ found a statistically significant shift in the distribution of mRS scores toward better outcomes in favor of EVT in the pooled analyses of 82 participants enrolled in 2 individual RCTs. However, EVT was not associated with improved 90-day functional outcomes in patients with ASPECTS of 3 or less in the RESCUE-Japan LIMIT trial.^7^ The reason for the different results of the RESCUE-Japan LIMIT trial could be explained in a few ways. First, the sample size in the RESCUE-Japan LIMIT trial was not large enough and the subanalysis was not prespecified. Second, the ASPECTS in the RESCUE-Japan LIMIT trial^7^ were detected and scored with diffusion-weighted imaging (DWI). ASPECTS estimation with DWI is known to overestimate the ischemic core compared with ASPECTS calculated with CT.^13,14^ The ASPECTS measured by DWI-MRI in patients with acute ischemic stroke has been reported as 1 scale lower than that measured by NCCT,^13^ Thus, the consistency of inclusion conditions will be affected. In our study, all of the ASPECTS were measured by NCCT, which is available at all stroke centers in China, so the consistency of the assessment criteria was maintained.

The results of subgroup analyses suggested that the benefit associated with EVT compared with SMT may have been greater among patients with lower baseline NIHSS score and shorter onset-to-imaging time. In a 2021 study^15^ assessing the association of EVT and NIHSS, patients with large artery occlusion and higher NIHSS scores generally had worse collateral circulation and experienced more severe symptoms. They were also more likely to receive a limited benefit in functional independence after intervention. High NIHSS scores were independent risk factors associated with poor 90-day outcomes.^15,16^ In terms of the association between EVT and time metrics, the odds of better disability outcomes at 90 days with the EVT group declined with longer time from symptom onset to arterial puncture. Each 1-hour delay to reperfusion was associated with less functional independence in a meta-analysis.^17^ Door-to-groin time was especially relevant for outcomes among patients at a comprehensive stroke center in Germany.^18^ In the RESCUE-Japan LIMIT trial, patients with last-known-well time and randomization longer than 6 hours had no statistically significant therapeutic outcome (OR, 2.49 [95% CI, 0.73-8.45)].^3^ These studies were similar to our results, which highlight that in patients with ASPECTS of 0 to 2, it is important to develop new methods to identify patients for more precisely tailored treatment.

In the ANGEL-ASPECT trial,^2^ patients with large ischemic strokes who underwent EVT had an 86% rate of thrombolysis in cerebral infarction reperfusion grade 2b or greater, and in the SELECT2 trial,^1^ the rate of successful reperfusion (thrombolysis in cerebral infarction ≥2b) was 79.8%. The successful reperfusion rate in large ischemic strokes was similar in our study. This may be due to higher thrombus loads and poor compensation of side branches in large ischemic strokes, which to some extent may have reduced successful revascularization.

In our study, the favorable functional outcome occurred in 22.2% of patients in the EVT group, which was similar to that of patients with ASPECTS of 3 or less in the RESCUE-Japan LIMIT trial (21.4%).^7^ The SMT group had favorable functional outcome in 9.9% of patients, lower than that reported in the RESCUE-Japan LIMIT trial in patients with ASPECTS of 3 or lower (18%).^7^ This is likely because there were only 8 patients with ASPECTS of 0 to 2 in the RESCUE-Japan LIMIT trial,^7^ while all of the patients in our study had ASPECTS of 0 to 2.

In our study, the rates of the sICH and any ICH of the patients with a very large ischemic core with ASPECTS of 0 to 2 were higher in the EVT group than the SMT group. The rate of any ICH in our study (EVT: 41.5% vs SMT: 11.4%) was similar to that reported in the ANGEL-ASPECT trial (EVT: 49.1% vs SMT: 17.3%) and SELECT2 trial (EVT: 58% vs SMT: 31%) and lower than the RESCUE-Japan LIMIT trial (EVT: 66.1% vs SMT: 32.0%). Rates of sICH were higher in the EVT group than the SMT group in our study (17.8% vs 0.9%), while there were no statistical differences in the RESCUE-Japan LIMIT trial of patients with the ASPECTS 3 to 5 or 3 or less, the ANGEL-ASPECT and SELECT2 RCTs of patients with the ASPECTS 3 to 5, or a cohort study of patients with ASPECTS of 2 to 5.^19^ These findings suggest that compared with the patients with ASPECTS of 3 to 5, patients with ASPECTS of 0 to 2 had higher risk of sICH after EVT. This may be because patients with low ASPECTS are at higher risk of sICH after EVT.^20,21^ Another potential explanation is that there were more patients with large artery atherosclerotic thrombosis in our study, which is associated with lower chance of successful reperfusion and a high number of thrombectomy attempts,^22,23^ and these patients usually need to receive antithrombotic medications. Although the sICH rate was higher in the EVT group in our study, there was no difference in mortality between groups. Clinicians may be hesitant to treat patients with ASPECTS of 0 to 2 with EVT because of the potential to save a patient’s life but leave them with very high disability (mRS score of 5). Our findings suggest that EVT would not expose large numbers of patients with stroke to more serious hazards nor convert patients who would otherwise have died into patients in a neurovegetative state, which many patients, their families, and clinicians consider worse than death.

### Limitations

Our study has several limitations. First, it has all the inherent limitations of a nonrandomized study. Some bias might be unavoidable, even with the PSM performed in our study. Second, the sample size in this study was not large enough for adequate analysis. Third, our sample only included patients in China, so the generalizability may be limited. The advantages of our study included its large-scale, prospective, multicenter design.

## Conclusions

This cohort study found that patients with very large ischemic core in NCCT within 24 hours of stroke onset had a higher odds of favorable functional outcome after EVT compared with SMT. Although the proportions of patients of sICH, any ICH, and cerebral hernia were higher in the EVT group than the SMT group, there were no statistically significant differences in mortality between groups. Part of the value of our study lies in that it found the same conclusions in medical practice as has been reported in RCTs.

## Notes

Supplement 1. **eTable 1.** Baseline Characteristics of Patients After PSM **eTable 2.** Primary, Secondary, and Safety Outcomes After PSM **eFigure.** Distribution of Modified Rankin Scale Score at 90 Days After PSM **eMethods.** Sample Size Calculation

Supplement 2.
Data Sharing Statement
