# Supplementary material for: Endovascular Therapy and Outcomes Among Patients With Very Large Ischemic Core Stroke
Source: JAMA Netw Open. 2024 May 2;7(5):e249298. doi: 10.1001/jamanetworkopen.2024.9298 (PMC11066696; doi:10.1001/jamanetworkopen.2024.9298)
Supplement: Supplement 1. — eTable 1. Baseline Characteristics of Patients After PSM eTable 2. Primary, Secondary, and Safety Outcomes After PSM eFigure. Distribution of Modified Rankin Scale Score at 90 Days After PSM eMethods. Sample Size Calculation [file jamanetwopen-e249298-s001.pdf]

Supplemental Online Content

Xie D, Huang J, Fan S, et al. Endovascular therapy and outcomes among patients with very large ischemic core stroke. *JAMA Netw Open*. 2024;7(5):e249298.
doi:10.1001/jamanetworkopen.2024.9298

eTable 1. Baseline Characteristics of Patients After PSM
eTable 2. Primary, Secondary, and Safety Outcomes After PSM
eFigure. Distribution of Modified Rankin Scale Score at 90 Days After PSM
eMethods. Sample Size Calculation

This supplemental material has been provided by the authors to give readers additional information about their work.

© 2024 Xie D et al. *JAMA Network Open.*

eTable 1

Baseline Characteristic	All	SMT (n=70)	EVT (n=70)	*P* Value
**Age, median (IQR), y**	70 (64, 78)	70 (65, 77)	70 (63, 79)	0.92
**Sex, No (%)**				
Female	70 (50)	36 (51.4)	34 (48.6)	0.74
Male	70 (50)	34 (48.6)	36 (51.4)	
**Medical history No. (%)**				
Diabetes	24 (17.1)	11 (15.7)	13 (18.6)	0.65
Hypertension	87 (62.1)	43 (61.4)	44 (62.9)	0.86
Atrial fibrillation	65 (46.4)	34 (48.6)	31 (44.3)	0.61
Hyperlipidemia	22 (15.7)	11 (15.7)	11 (15.7)	>0.99
Smoking	28 (20.0)	12 (17.1)	16 (22.9)	0.40
**Clinical characteristics**				
Systolic BP, mmHg, median (IQR)	150 (132, 170)	151 (130, 178)	150 (137, 170)	0.91
Diastolic BP, mmHg, median (IQR)	87 (76, 101)	86 (78, 100)	89.5 (72, 102)	0.95
Hemisphere, No. (%)				
left	69 (49.3)	34 (48.6)	35 (50.0)	0.87
right	71 (50.0)	36 (51.0)	35 (50.0)	
Glucose, mmol/L, median (IQR)	7.3 (6.2, 8.6)	7.3 (6.0, 8.1)	7.5 (6.3, 9.1)	0.25
IVT, No. (%)	40 (28.6)	21 (30)	19 (27.1)	0.71
General Anesthesia, No. (%)	NA	NA	12 (17.1)	NA
Baseline NIHSS score, median (IQR)	18 (15, 24)	18 (14, 25)	18 (16, 23)	0.62
Baseline ASPECTS, median (IQR)				
0-1	98 (70)	49 (70)	49 (70)	>0.99
2	42 (30)	21 (30)	21 (30)	
**Onset to imaging (minutes) (IQR)**	314 (169.5, 527.8)	299.5 (175, 503.8)	364 (147.3, 585.3)	0.74
**Occlusion site No. (%)**				
Intracranial internal carotid artery	70 (50.0)	35 (50.0)	35 (50.0)	0.77
M1 middle cerebral artery segment	62 (44.3)	32 (45.7)	30 (42.9)	
M2 middle cerebral artery segment	8 (5.7)	3 (4.3)	5 (7.1)	
**Stroke causative mechanism No. (%)**				
LAA	44 (31.4)	22 (31.4)	22 (31.4)	0.86

© 2024 Xie D et al. *JAMA Network Open.*

CE	80 (57.1)	41 (58.6)	39 (55.7)	
Other	1 (1.4)	3 (1.4)	3 (1.4)	
Unknown	12 (8.6)	6 (8.6)	6 (8.6)	
**Successful recanalization mTICI ≥2b, No. (%)**	NA	NA	59 (84.2)	NA

eTable 2

Outcomes	SMT (n=70)	EVT (n=70)	*P* value	Unadjusted OR, *P*	Adjusted OR#, *P*
Primary Outcome					
mRS0-3 (%)	6 (8.6)	17 (24.3)	0.01	3.42 (1.26-9.29), 0.02	5.00 (1.63-15.27), 0.005
Secondary Outcomes					
mRS0-1 (%)	1 (1.4)	0 (0)	0.32	NA	NA
mRS0-2 (%)	1 (1.4)	9 (12.9)	0.009	10.18 (1.25-82.68), 0.03	27.75 (2.5-308.06) 0.007
mRS=5 (%)	12(17.1)	5 (7.1)	0.07	0.37 (0.12-1.12), 0.08	0.32 (0.10-1.00) 0.05
Safety Outcomes					
sICH (%)	1 (1.4)	7 (10)	0.03	7.67 (0.92-64.06) 0.06	8.91 (0.98-81.20) 0.05
Any ICH (%)	10 (14.3)	22 (31.4)	0.02	2.75 (1.19-6.36), 0.02	2.94 (1.24-6.94) 0.01
Cerebral hernia	16 (22.9)	33 (47.1)	0.003	3.01 (1.45-6.24) 0.003	3.32 (1.51-7.28) 0.003
Mortality (%)	37 (52.9)	41 (58.6)	0.50	1.26 (0.65-2.46), 0.50	1.30 (0.62-2.72), 0.49

Adjusting for age, baseline NIHSS, IVT, onset to imaging, stroke causative mechanism

© 2024 Xie D et al. *JAMA Network Open.*

eFigure

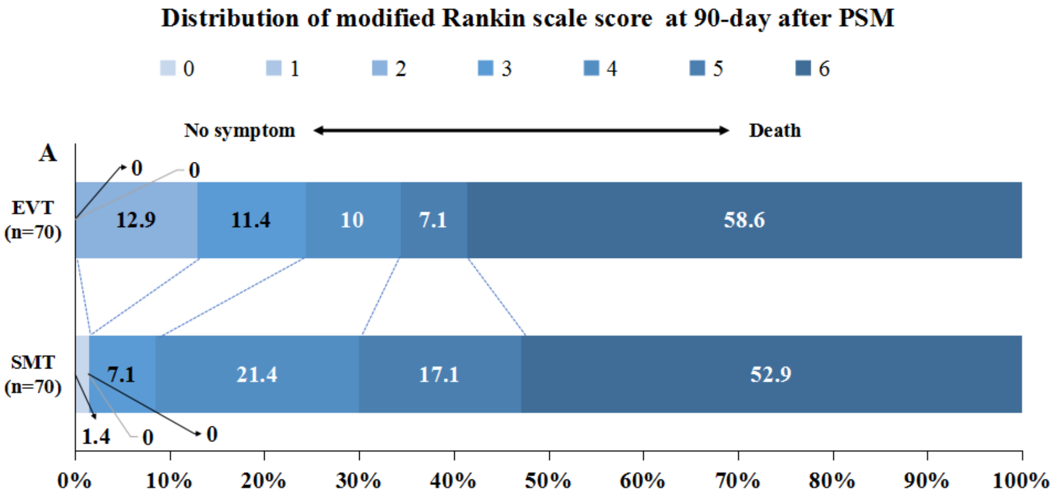

© 2024 Xie D et al. *JAMA Network Open.*

eMethods

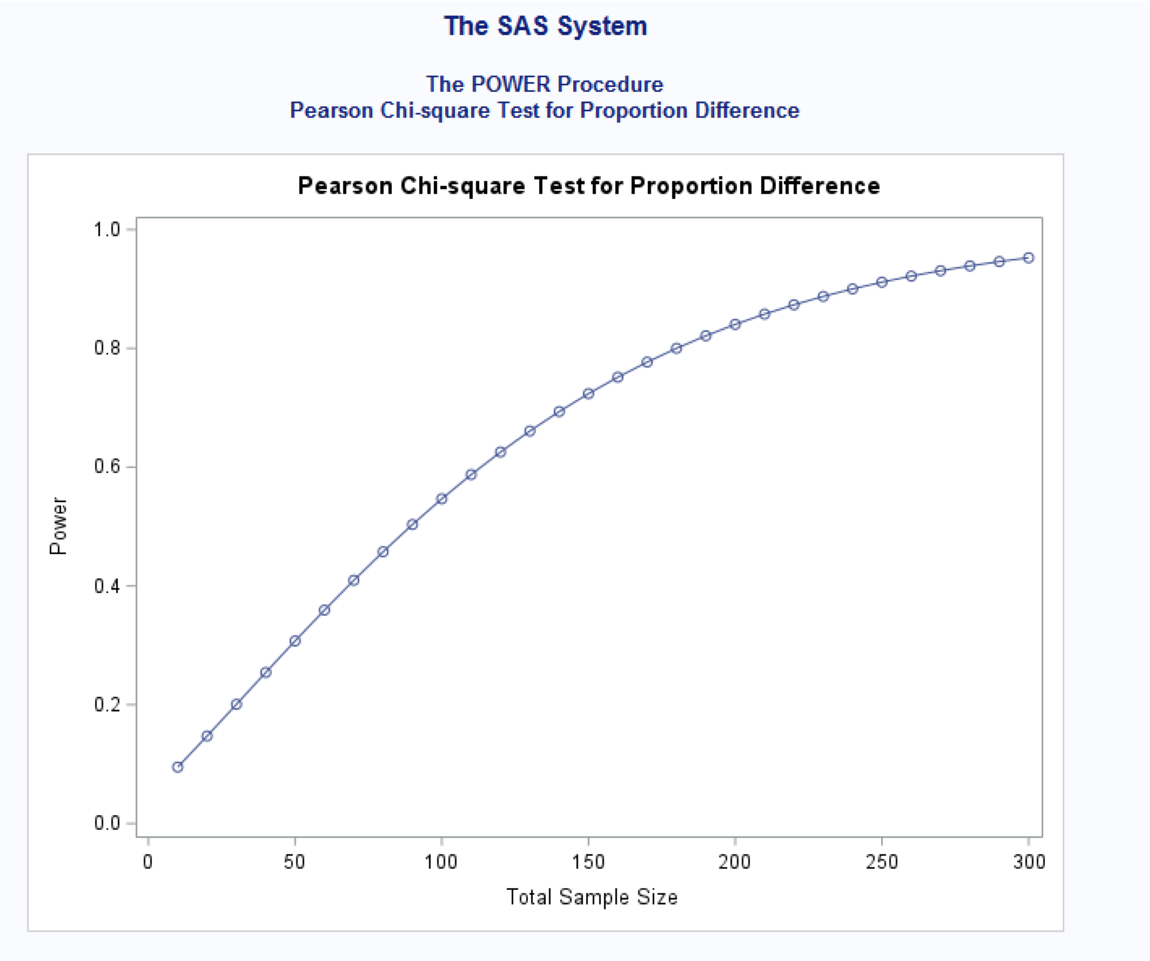

We assume the rate of achieving a favorable outcome for patients with ASPECTS of 0 to 2 was 0.267 in the SMT group, and assuming an 20% treatment effect of EVT. The Figure illustrates how the power of the study changes with different total sample sizes, maintaining a ratio of EVT:SMT = 1:1 and using a two-sided alpha level of 0.05. Based on these calculations, it was found the current sample size would provide a power greater than 0.904 with a two-sided alpha level of 0.05.

Moreover, the sample size of current study is three-fold than the meta-analysis the subgroup of patients with a baseline ASPECTS of lower than 2 containing 2 randomized trials, and is the largest sample size study which compared the EVT and SMT in patients with extended large core infarction.

© 2024 Xie D et al. *JAMA Network Open.*
