# Supplementary material for: Endovascular Therapy and Outcomes Among Patients With Very Large Ischemic Core Stroke
Source: JAMA Netw Open. 2024 May 2;7(5):e249298. doi: 10.1001/jamanetworkopen.2024.9298 (PMC11066696; doi:10.1001/jamanetworkopen.2024.9298)
Supplement: Supplement 2. — Data Sharing Statement [file jamanetwopen-e249298-s002.pdf]

Data Sharing Statement

Xie. Endovascular Therapy and Outcomes Among Patients With Very Large Ischemic Core Stroke. *JAMA Netw Open*. Published May 02, 2024. doi:10.1001/jamanetworkopen.2024.9298

Data
Data available: No
